# Subsite-specific benefit of induction chemoimmunotherapy in HPV-negative oropharyngeal squamous cell carcinoma

**DOI:** 10.3389/fonc.2026.1861897

**Published:** 2026-06-11

**Authors:** Lin Zhu, Die Zhang, Jie Chen, Wei Qian, Peiyao Liu, Chunying Shen, Tingting Xu, Xin Zhou, Xueguan Lu

**Affiliations:** 1 Department of Radiation Oncology, Fudan University Shanghai Cancer Center, Shanghai, China; 2 Department of Oncology, Shanghai Medical College, Fudan University, Shanghai, China; 3 Shanghai Clinical Research Center for Radiation Oncology, Shanghai, China; 4 Shanghai Key Laboratory of Radiation Oncology, Shanghai, China

**Keywords:** definitive chemoradiotherapy, HPV-negative oropharyngeal squamous cell carcinoma, immune checkpoint inhibitors, induction chemotherapy, primary tumor subsite

## Abstract

**Purpose:**

To evaluate whether the addition of immune checkpoint inhibitors (ICIs) to induction chemotherapy improves outcomes in HPV-negative oropharyngeal squamous cell carcinoma (OPSCC), and whether efficacy differs by primary tumor subsite.

**Methods:**

We retrospectively analyzed 99 patients with newly diagnosed HPV-negative OPSCC treated with induction therapy followed by definitive chemoradiotherapy. Patients received induction chemotherapy alone (IC; n = 73) or IC plus ICI (n = 26). Progression-free survival (PFS) was the primary end point and overall survival (OS) was secondary. Propensity score matching (PSM) was used to adjust for baseline imbalances. Subgroup analyses were performed for base-of-tongue (BOT) and non-BOT tumors.

**Results:**

In the overall cohort, IC+ICI was associated with numerically improved PFS and OS, but differences were not statistically significant before or after PSM. In the non-BOT cohort, IC+ICI was associated with significantly improved PFS both before and after PSM. Before matching, 24- and 36-month PFS rates were 100% and 100% with IC+ICI versus 74.0% and 68.9% with IC; after matching, the corresponding rates were 100% and 100% versus 77.0% and 72.9%, respectively. OS did not significantly differ, although outcomes consistently favored IC+ICI. In the BOT cohort, neither PFS nor OS differed significantly between groups.

**Conclusion:**

In HPV-negative OPSCC, adding ICI to induction chemotherapy showed numerically favorable but statistically non-significant PFS and OS differences in the overall cohort. Exploratory subgroup analyses suggested a potential immunotherapy-related benefit in patients with non-BOT primary tumors, warranting prospective validation.

## Introduction

Head and neck squamous cell carcinoma (HNSCC) is among the most common cancers worldwide, the incidence of which continues to rise and is projected to increase by 30% by 2030 (1). The burden of HNSCC varies across countries/regions and has generally been correlated with exposure to tobacco-derived carcinogens, excessive alcohol consumption, or both. Oropharyngeal squamous cell carcinoma (OPSCC) is an HNSCC subtype affecting the oropharynx, which includes the tonsillar regions, the base of tongue, the soft palate and posterior pharyngeal wall (2). Recent increases in incidence have been increasingly driven by the rising prevalence of virus-related disease, particularly HPV-associated oropharyngeal cancer, whereas the incidence of tobacco- and alcohol-related tumors has declined in many populations (3).

Based on etiology, OPSCC can be broadly classified into HPV(human papillomavirus) -negative (alcohol and tobacco induced) and HPV - positive (HPV infection)disease, which are generally diagnosed by an over 70% expression of the p16 protein (p16 +) (4). HPV- positive OPSCC is more sensitive to radiotherapy and chemotherapy and is associated with significantly improved survival compared with HPV- negative OPSCC (5). In contrast, HPV-negative OPSCC is associated with a more aggressive clinical course, poorer prognosis, and higher resistance to therapy (6). Despite advances in multimodal treatment, outcomes for HPV-negative OPSCC remain unsatisfactory (7). The 5-year overall survival (OS) rate is approximately 47%–54%, and up to half of patients experience recurrence and distant metastasis after standard therapy (8). Therefore, improving outcomes in patients with HPV-negative OPSCC remains an important clinical challenge.

In locally advanced HNSCC, induction chemotherapy has not consistently improved survival over standard concurrent chemoradiotherapy in randomized studies (9, 10). Over the past decade, immune checkpoint inhibitors (ICIs) targeting the PD-1/PD-L1 axis have become established treatments for recurrent or metastatic HNSCC. In particular, clinical studies of anti-PD-1 agents such as nivolumab and pembrolizumab have demonstrated survival benefit compared with standard regimens in selected patients with recurrent or metastatic disease (11–15). PD-1 blockade combined with platinum-based chemotherapy has also improved first-line outcomes in recurrent/metastatic HNSCC and is now an important standard treatment option (12). These findings have supported the evaluation of ICIs in earlier disease settings. Preclinical and clinical studies suggest that neoadjuvant immunotherapy may enhance antitumor activity, reduce high-risk pathological features, and promote tumor downstaging (11, 16–18). More recently, perioperative immunotherapy demonstrated clinical benefit in resectable locally advanced HNSCC in the phase III KEYNOTE-689 study (19). However, for patients treated with definitive radiotherapy or chemoradiotherapy, the role of neoadjuvant or induction PD-1 blockade, with or without chemotherapy, remains uncertain, and large phase III trials adding ICIs to definitive chemoradiotherapy have not established a clear survival benefit (20, 21).

The anti-PD-1 agent used in the present study was toripalimab, a recombinant humanized immunoglobulin G4 monoclonal antibody against PD-1. Toripalimab binds to PD-1 and blocks its interaction with PD-L1 and PD-L2, thereby restoring antitumor T-cell activity (22, 23). Toripalimab was initially developed and approved in China and has been most extensively studied in tumors with high prevalence in Asian populations, particularly nasopharyngeal carcinoma (23, 24). Recently, early-phase studies have also explored toripalimab-based neoadjuvant chemoimmunotherapy in locally advanced HNSCC, reporting encouraging response rates and acceptable toxicity (25, 26). Therefore, the role of toripalimab-containing induction chemoimmunotherapy before definitive RT/CCRT in HPV-negative OPSCC warrants further investigation.

Based on these considerations, we performed this study to analyze the outcomes of HPV-negative OPSCC patients treated with induction chemotherapy combined with immune checkpoint inhibitors before IMRT to the primary disease, with the aim of exploring this therapeutic strategy and identifying relevant prognostic factors.

## Patients and methods

### Patients and selection criteria

This retrospective study included 99 patients with human papillomavirus (HPV)-negative oropharyngeal squamous cell carcinoma (OPC) who received radiotherapy with or without chemotherapy at Fudan University Shanghai Cancer Center between January 2017 and April 2025.

The inclusion criteria were as follows: (1) age ≥18 years; (2) newly diagnosed, pathologically confirmed squamous cell carcinoma of the oropharynx, with HPV-negative status defined as either negative p16 expression by immunohistochemistry or negative HPV by polymerase chain reaction (PCR); clinical stage T1–4, N0–3, M0 according to the 8th edition of the American Joint Committee on Cancer (AJCC) staging system; (3) Eastern Cooperative Oncology Group (ECOG) performance status ≤1; and (4) receipt of induction therapy (chemotherapy with or without ICI) followed by chemoradiation therapy (CRT), consisting of definitive intensity-modulated radiotherapy (IMRT) to the primary tumor with concurrent chemotherapy. Concurrent chemotherapy consisted of intravenous cisplatin at 80 mg/m², administered over 3 days every 3 weeks, for a total of 2 cycles during CRT.

This study was approved by the Institutional Review Board of Fudan University Shanghai Cancer Center. All procedures were conducted in accordance with institutional ethical standards and the Declaration of Helsinki (1964) and its subsequent amendments.

### Treatment protocol

Induction therapy: A total of 73 patients received induction chemotherapy (IC group). The induction regimen consisted of docetaxel (75 mg/m², day 1) plus cisplatin (25 mg/m², days 1–3) administered every 21 days for two cycles, followed by definitive radiotherapy. Thirty-six patients received induction chemotherapy combined with immunotherapy (IC+ICI group). The regimen consisted of docetaxel (75 mg/m², day 1) plus cisplatin (25 mg/m², days 1–3) combined with toripalimab (240 mg, day 1), administered every 21 days for two cycles, followed by definitive radiotherapy.

CRT: All patients underwent IMRT after completion of induction therapy. Patients were immobilized in the supine position using a thermoplastic mask. Contrast-enhanced T1-weighted magnetic resonance imaging (MRI) was co-registered with planning computed tomography (CT) scans for target delineation, including the gross tumor volume (GTV). Based on cTNM staging, the prescribed dose to the planning target volume (PTV) encompassing the primary tumor and involved cervical lymph nodes ranged from 66 to 70 Gy, delivered in 33–35 fractions. Concurrent chemotherapy with cisplatin was administered for two cycles during radiotherapy.

### Statistical analysis

All statistical analyses were performed using R software (version 4.5.1) and SPSS (version 22.0; IBM Corp.). Progression-free survival (PFS) was defined as the time from the date of diagnosis (or initiation of treatment) to the first documented disease progression, recurrence, or death from any cause, whichever occurred first. Overall survival (OS) was defined as the time from diagnosis (or treatment initiation) to death from any cause. Survival outcomes were estimated using the Kaplan–Meier method and compared using the log-rank test. Prognostic factors were evaluated using univariable and multivariable Cox proportional hazards regression models, with hazard ratios (HRs) and 95% confidence intervals (CIs) reported. To reduce potential selection bias and balance baseline characteristics between the IC and IC+ICI groups, propensity score matching (PSM) was performed using a nearest-neighbor algorithm with a 1:1 matching ratio, without replacement, and a caliper width of 0.02. Covariates included age, sex, clinical T stage, clinical N stage, and overall clinical stage. Standardized mean differences (SMDs) were used to assess covariate balance before and after matching. Comparisons of clinicopathological characteristics between groups were conducted using the chi-square (χ²) test for categorical variables, the independent-samples t-test for normally distributed continuous variables, and the Wilcoxon rank-sum test for non-normally distributed variables. All statistical tests were two-sided, and a p-value <0.05 was considered statistically significant.

## Results

### Patient characteristics

A total of 99 patients with histologically confirmed HPV-negative OPSCC were included in the study (**Table 1**). The mean age was 59.19 ± 8.58 years, and most patients were male (90.9%). Never smokers accounted for 66.7% of the cohort, with a mean smoking exposure of 9.42 ± 18.12 pack-years. The tonsillar region was the most common primary site (60.6%), followed by the base of tongue (BOT; 29.3%), soft palate (7.1%), and posterior pharyngeal wall (3.0%). Most patients had locally advanced disease, including 46.5% with stage IVa and 25.3% with stage IVb disease. Nodal involvement was common, with 43.4% classified as N2 and 23.2% as N3. Regarding induction treatment, 73.7% received induction chemotherapy alone and 26.3% received induction chemotherapy plus ICIs.

**Table 1:** Baseline clinical characteristics of the study population.

Characteristic	Level	Overall (n=99)
Age, mean ± SD		59.19 ± 8.58
Sex, n (%)	Male	90 (90.9)
	Female	9 (9.1)
Smoking Status, n (%)	Never smoker	66 (66.7)
	Current/Former smoker	33 (33.3)
Pack-years, mean ± SD		9.42 ± 18.12
Primary Site, n (%)	Tonsillar region	60 (60.6)
	Base of tongue (BOT)	29 (29.3)
	Soft palate	7 (7.1)
	Posterior pharyngeal wall(PPW)	3 (3.0)
cT Stage, n (%)	T1	18 (18.2)
	T2	35 (35.4)
	T3	21 (21.2)
	T4a	23 (23.2)
	T4b	2 (2.0)
cN Stage, n (%)	N0	1 (1.0)
	N1	32 (32.3)
	N2	43 (43.4)
	N3	23 (23.2)
cTNM Stage (AJCC 8th), n (%)	III	28 (28.3)
	IVa	46 (46.5)
	IVb	25 (25.3)
Induction Therapy, n (%)	Chemotherapy	73 (73.7)
	Chemotherapy + ICI	26 (26.3)

SD, standard deviation; BOT, base of tongue; PPW, Posterior pharyngeal wall; AJCC, American Joint Committee on Cancer; ICI, immune checkpoint inhibitors; n, number of patients; cTNM Stage, clinical TNM stage before treatment.

### Response and survival outcomes in the overall cohort

Response data are summarized in **Supplementary Table 1**. The overall response rate(ORR) was high in both groups and did not differ significantly between IC and IC+ICI (86.0% vs 82.6%; P = .736). Although 1 complete response was observed in the IC+ICI group, overall radiographic response was broadly comparable between groups.

**Figure 1:**
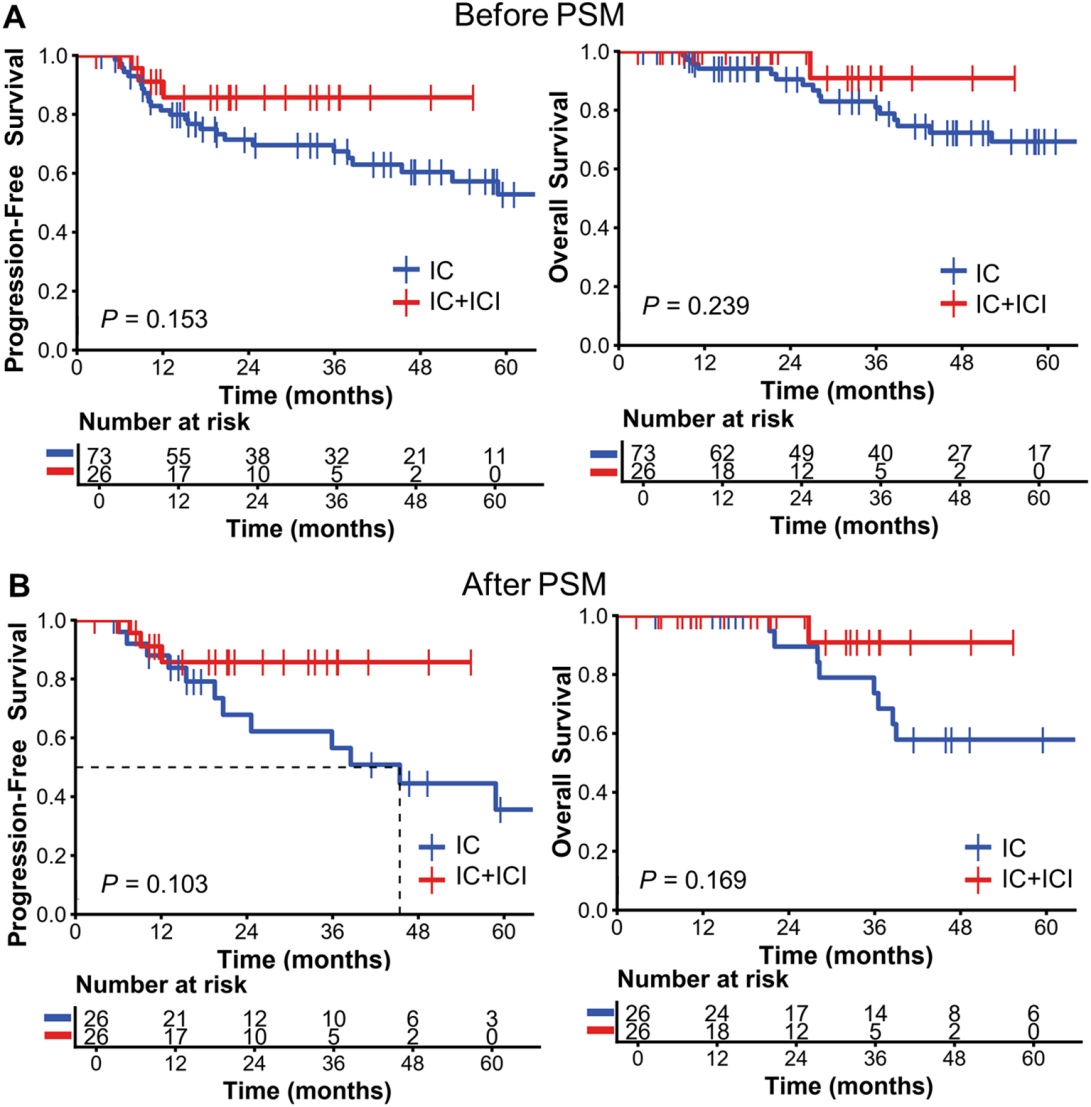
Kaplan–Meier survival analysis for progression-free survival (PFS) and overall survival (OS) in the overall cohort. **(A)** PFS and OS according to induction treatment group before propensity score matching (PSM), comparing induction chemotherapy (IC) alone with IC plus immune checkpoint inhibitor (IC+ICI). **(B)** PFS and OS according to induction treatment group after PSM.

In the overall cohort, the median follow-up duration was 37.8 months (95% CI, 32.6 - 49.3 months). Before PSM, 73 patients received IC alone and 26 received IC+ICI. Baseline characteristics were generally similar, although some imbalance remained for cN stage and cTNM stage. After PSM, 26 patients were retained in each group, with improved covariate balance (**Table 2**). PFS tended to be better in the IC+ICI group both before and after matching, but the difference was not statistically significant (**Figure 1**). Before PSM, the 24- and 36-month PFS rates were 85.7% and 85.7% with IC+ICI, compared with 71.4% and 67.4% with IC alone (log-rank P = .153). After PSM, the corresponding rates were 85.7% and 85.7% versus 67.8% and 56.5% (log-rank P = .103). A similar pattern was seen for OS. Before PSM, the 24- and 36-month OS rates were 100% and 90.9% in the IC+ICI group, versus 90.5% and 80.9% in the IC group (log-rank P = .239); after PSM, they were 100% and 90.9% versus 89.5% and 73.7%, respectively (log-rank P = .169). Overall, the addition of ICI was associated with a numerical improvement in survival, although no significant benefit was observed in the overall cohort.

**Table 2:** Characteristics of all patients treated with induction therapy and PSM analysis.

Characteristic	Before PSM				After PSM			
	IC	IC+ICI	*P*	SMD^1^	IC	IC+ICI	*P*	SMD^1^
	n= 73 (%)	n= 26 (%)			n= 26 (%)	n= 26 (%)		
Age^2^	58.99 ± 8.47	59.77 ± 9.04	0.692	0.089	60.58 ± 8.37	59.77 ± 9.04	0.74	0.093
Gender			0.914	0.111			0.603	0.292
Female	6 (8.2)	3 (11.5)			1 (3.8)	3 (11.5)		
Male	67 (91.8)	23 (88.5)			25 (96.2)	23 (88.5)		
cT Stage			0.309	0.285			0.764	0.167
T1-3	57 (78.1)	17 (65.4)			19 (73.1)	17 (65.4)		
T4	16 (21.9)	9 (34.6)			7 (26.9)	9 (34.6)		
cN Stage			0.061	0.472			1.000	<0.001
N1-2	60 (82.2)	16 (61.5)			16 (61.5)	16 (61.5)		
N3	13 (17.8)	10 (38.5)			10 (38.5)	10 (38.5)		
cTNM Stage			0.148	0.418			1.000	<0.001
III	24 (32.9)	4 (15.4)			4 (15.4)	4 (15.4)		
IV	49 (67.1)	22 (84.6)			22 (84.6)	22 (84.6)		

Non-BOT, non-base-of-tongue; IC, Induction Chemotherapy; IC+ ICI, Induction Chemotherapy + ICI; SMD, Standardized Mean Difference. ^1^Imbalance between treatment groups was defined as a SMD ≥0.1; balance between treatment groups was defined as a SMD <0.1. ^2^Variables were described by mean ± SD.

### Factors associated with progression-free survival

Univariable and multivariable Cox regression analyses for PFS are shown in **Table 3**. In univariable analysis, induction chemotherapy plus ICI showed a nonsignificant trend toward improved PFS compared with chemotherapy alone (HR, 0.43; 95% CI, 0.13 to 1.42; P = .165). BOT primary tumors showed a nonsignificant trend toward worse PFS relative to tonsillar tumors (HR, 1.75; 95% CI, 0.80 to 3.82; P = .164), and cT4 disease was also associated with numerically poorer PFS compared with T1 disease (HR, 1.58; 95% CI, 0.56 to 4.45; P = .390). In multivariable analysis, no factor remained independently associated with PFS. BOT tumors continued to demonstrate a nonsignificant trend toward shorter PFS versus tonsillar tumors (HR, 1.77; 95% CI, 0.78 to 4.02; P = .172), whereas induction chemotherapy plus ICI continued to show a nonsignificant trend toward improved PFS compared with chemotherapy alone (HR, 0.36; 95% CI, 0.10 to 1.32; P = .124).

**Table 3:** Univariate and multivariate cox regression analysis for PFS risk factors.

Risk factor	Univariate analysis		Multivariate analysis	
	HR (95% CI)	*P* value	HR (95% CI)	*P* value
Age	1.03 (0.98–1.07)	0.275	1.03 (0.98–1.08)	0.322
Pack-years	1.01 (0.99–1.03)	0.321	1.01 (0.99–1.03)	0.189
Sex				
Female	1 (Ref)	–	–	–
Male	3.98 (0.54–29.43)	0.176	–	–
cTNM Stage (AJCC 8th)				
III	1 (Ref)	–	1 (Ref)-	–
IVa	1.12 (0.49–2.56)	0.787	1.41 (0.58–3.43)	0.451
IVb	0.68 (0.23–2.03)	0.488	0.93 (0.29–2.95)	0.897
cT Stage				
T1	1 (Ref)	–	–	–
T2	0.56 (0.19–1.67)	0.299	–	–
T3	1.29 (0.43–3.86)	0.644	–	–
T4	1.58 (0.56–4.45)	0.39	–	–
cN Stage				
N1	1 (Ref)	–	–	–
N2	0.86 (0.39–1.93)	0.718	–	–
N3	0.65 (0.23–1.87)	0.422	–	–
Primary Site				
Tonsil	1 (Ref)	–	1 (Ref)	–
BOT	1.75 (0.80–3.82)	0.164	1.77 (0.78–4.02)	0.172
Soft palate and PPW	1.28 (0.37–4.44)	0.693	1.16 (0.32–4.15)	0.823
Induction Therapy				
Chemotherapy	1 (Ref)		1 (Ref)	
Chemotherapy + ICI	0.43 (0.13–1.42)	0.165	0.36 (0.10–1.32)	0.124

BOT, base of tongue; PPW, Posterior pharyngeal wall; HR, Hazard Ratio; CI, Confidence Interval; Ref, Reference group; AJCC, American Joint Committee on Cancer; cTNM Stage, clinical TNM stage before treatment.

### Baseline characteristics in the non-BOT and BOT cohorts

Because exploratory analyses suggested subsite-specific differences in survival, subgroup analyses were performed according to primary tumor subsite (non-BOT v BOT). Patients with BOT tumors were older overall than those with non-BOT tumors, but the 2 cohorts were otherwise similar in baseline clinicopathologic characteristics. Within the non-BOT cohort, baseline characteristics were generally balanced between the IC and IC+ICI groups and were further balanced after PSM. Within the BOT cohort, treatment groups were also broadly comparable, although some baseline imbalance in nodal stage was observed before matching. After PSM, overall covariate balance improved in both subgroups, with slight residual age imbalance in the BOT cohort (**Supplementary Tables 2**, **S3**).

### Survival outcomes according to primary tumor subsite

**Figure 2:**
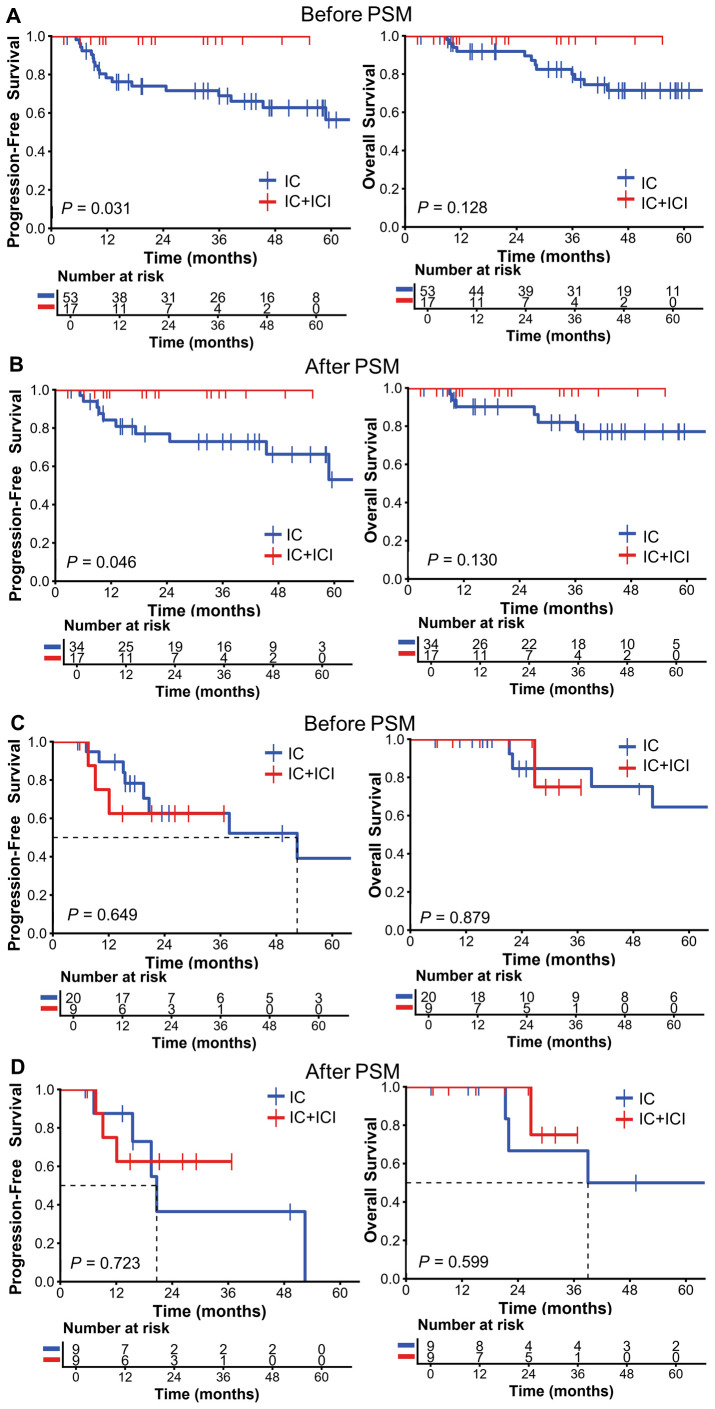
Kaplan–Meier survival analysis by primary tumor sites and treatment groups. **(A)** Progression-free survival (PFS) and overall survival (OS) curves for patients with non–base-of-tongue (non-BOT) primary tumor sites receiving induction chemotherapy (IC) or induction chemotherapy combined with immunotherapy (IC+ICI, with or without targeted therapy) before propensity score matching (PSM). **(B)** PFS and OS curves for the non-BOT cohort after PSM. **(C)** PFS and OS curves for patients with base-of-tongue (BOT) primary tumors before PSM. **(D)** PFS and OS curves for the BOT cohort after PSM.

In the non-BOT cohort, adding ICI to induction chemotherapy was associated with significantly improved PFS both before and after PSM (**Figures 2A, B**). Before matching, the 24- and 36-month PFS rates were 100% in the IC+ICI group versus 74.0% and 68.9%, respectively, in the IC group (log-rank P = .031). This separation remained after matching, with corresponding 24- and 36-month PFS rates of 100% and 100% in the IC+ICI group compared with 77.0% and 72.9% in the IC group (log-rank P = .046). By contrast, OS was not significantly different between groups, although outcomes consistently favored IC+ICI. Before PSM, the 24- and 36-month OS rates were 100% and 100% with IC+ICI versus 91.9% and 79.9% with IC alone (log-rank P = .128); after PSM, the corresponding rates were 100% and 100% versus 90.2% and 82.0%, respectively (log-rank P = .130).

In contrast, no significant differences in either PFS or OS were observed between treatment groups in the BOT cohort, either before or after PSM (**Figures 2C, D**). Before matching, 24- and 36-month PFS rates were nearly identical between groups, at 62.6% in the IC group and 62.5% in the IC+ICI group (log-rank P = .649). OS likewise did not differ significantly, with 24- and 36-month rates of 84.6% and 84.6% in the IC group versus 100% and 75.0% in the IC+ICI group (log-rank P = .879). After matching, the 24- and 36-month PFS rates were 36.5% and 36.5% in the IC group and 62.5% and 62.5% in the IC+ICI group (log-rank P = .723), whereas the corresponding OS rates were 66.7% and 66.7% versus 100% and 75.0%, respectively (log-rank P = .599).

## Discussion

In the overall cohort, the addition of ICI to induction chemotherapy was associated with numerically longer PFS and OS, although these differences were not statistically significant. Exploratory subgroup analyses suggested that this signal may not be evenly distributed across HPV-negative OPSCC. A nominal PFS benefit favoring IC+ICI was observed in patients with non-BOT primary tumors, whereas no comparable signal was observed in the BOT cohort. Because these subgroup analyses were exploratory, the non-BOT finding should be interpreted cautiously and viewed as hypothesis-generating. Taken together, these results suggest that the benefit of induction chemoimmunotherapy may vary by primary tumor subsite and primary tumor subsite may warrant further investigation in future prospective studies of induction chemoimmunotherapy in HPV-negative OPSCC.

HPV-negative OPSCC is generally associated with less favorable outcomes than HPV-positive disease, which has driven continued efforts to improve upon conventional chemoradiotherapy. ICIs have already reshaped the standard-of-care landscape in recurrent or metastatic HNSCC (27). However, translating this benefit into the curative-intent setting has proved more difficult. Early phase 3 studies evaluating concurrent immune checkpoint blockade with definitive CRT were negative (28). In JAVELIN Head and Neck 100, the addition of avelumab to cisplatin-based CRT failed to improve survival (29), and KEYNOTE-412 likewise did not show a significant survival benefit with pembrolizumab given concurrently with CRT (30). Similarly, adjuvant or maintenance immunotherapy after definitive multimodality treatment has not yet produced a clear survival gain in unselected populations, as illustrated by the negative IMvoke010 trial (31). Smaller randomized trials also failed to show benefit for concurrent immune checkpoint blockade with CRT (32–34). However, in a randomized phase II study comparing concurrent versus sequential pembrolizumab with chemoradiation in locally advanced HNSCC, the sequential arm showed better 1-year PFS and more favorable long-term locoregional control (35). These results suggest that simply adding immunotherapy to standard CRT may be insufficient, and that treatment timing, patient selection, and disease context are likely to matter.

At the same time, the broader curative-intent literature should not be interpreted as uniformly negative. In resectable locally advanced HNSCC, perioperative pembrolizumab in KEYNOTE-689 improved event-free survival and increased major pathologic response rates, supporting the idea that earlier integration of immunotherapy may benefit selected patients (19). In unresected disease, data on induction chemoimmunotherapy remain comparatively limited, but the phase 2 DEPEND study showed that neoadjuvant nivolumab plus chemotherapy could induce deep responses in a substantial proportion of patients with HPV-negative locally advanced HNSCC, with encouraging survival outcomes (2-year PFS and OS of 66% and 73%, respectively) after response-adapted CRT (28). Although that study included multiple head and neck subsites and was not designed to address OPSCC subsite-specific effects, it supports the biological and clinical plausibility of an induction immunotherapy-based strategy. Ongoing phase 3 studies, including JADE and eVOLVE-HNSCC, further reflect continued interest in optimizing the sequential use of immunotherapy after definitive CRT in unresected locally advanced disease (36, 37).

Against this background, the most clinically relevant observation in our study was the consistent PFS advantage seen in the non-BOT subgroup. This pattern was present before and after propensity score matching. By contrast, in the overall cohort, the effect of IC plus ICI was directionally favorable but modest, and in the BOT subgroup no clear benefit emerged. Our results therefore support the possibility that primary tumor subsite modifies the apparent effect of induction chemoimmunotherapy in HPV-negative OPSCC. This point is clinically important because BOT and non-BOT tumors are often grouped together within OPSCC despite meaningful differences in anatomy, presentation, and possibly treatment sensitivity.

Several factors may have contributed to the subsite-specific pattern observed in this study, although these explanations remain hypothesis-generating. First, tumor–immune interactions may not be uniform across oropharyngeal subsites. Prior studies have reported differences in immune-cell infiltration and clinicobiologic features between tonsillar and BOT tumors, including variation in CD8+ T cells, CD20+ B cells, CD68+ macrophages, and CD163+ macrophages (38, 39). These findings raise the possibility that local immune contexture may differ by subsite. Second, BOT tumors often present with more advanced locoregional disease, which may be associated with greater tumor burden, hypoxia, and a less favorable treatment microenvironment (40, 41). Third, local treatment factors may also be relevant. The infiltrative growth pattern of BOT tumors, limited anatomic boundaries, and motion related to swallowing or tongue-base displacement may increase uncertainty in target definition and dose delivery (42). In HPV-negative disease, where radiosensitivity is generally lower than in HPV-positive tumors, these anatomic and biologic challenges may be particularly important (43, 44). Taken together, the inferior outcomes observed in BOT primaries are likely multifactorial, potentially reflecting adverse local anatomy, heavier locoregional disease burden, and biologic differences in treatment responsiveness.

Beyond subsite heterogeneity, another finding was the discrepancy between radiographic response and survival outcome. Notably, the similar ORR but numerically improved PFS with IC+ICI suggests that early radiographic response after induction therapy may not fully capture the clinical effect of ICI. RECIST-based ORR mainly reflects changes in tumor burden, whereas immunotherapy benefit may be reflected more by durable disease control than by a higher initial response rate (45). This interpretation is consistent with prior work showing that conventional RECIST may incompletely characterize atypical or delayed response patterns during immunotherapy, which led to the development of immune-adapted response criteria such as iRECIST (46, 47).

These findings have potential implications for future trial design. Our data do not support viewing HPV-negative OPSCC as a fully homogeneous disease when evaluating induction immunotherapy-based approaches. Instead, non-BOT tumors may represent a subgroup more likely to derive benefit, whereas BOT tumors may require different selection criteria, alternative intensification strategies, or more refined biologic stratification. Prospective studies should therefore consider stratifying by primary tumor subsite at enrollment rather than treating BOT and non-BOT tumors as interchangeable categories within HPV-negative OPSCC.

### Limitations

This study has several limitations. First, its retrospective design introduces the possibility of selection bias and residual confounding despite the use of propensity score matching. Second, the sample size was limited, particularly in the subgroup analyses, which reduces statistical power and makes negative findings difficult to interpret with confidence. Moreover, treatment-by-subsite interaction analyses did not detect a statistically significant interaction for PFS or OS before or after PSM, although these analyses were limited by sparse events, including no PFS events in the non-BOT IC+ICI subgroup. Therefore, the observed non-BOT PFS signal should be interpreted as hypothesis-generating. Third, all patients in this cohort received induction-based treatment followed by definitive CCRT; therefore, we were unable to compare this strategy directly with upfront standard CCRT. In addition, detailed toxicity data were not consistently available from the retrospective medical records, and adverse events were not uniformly graded according to CTCAE criteria. Therefore, safety outcomes were not included in the present analysis. Finally, key biologic correlates, including PD-L1 expression and other immune microenvironmental markers, were not available, which limits mechanistic interpretation and prevents more refined biomarker-based patient selection. Future studies should incorporate prospective validation, larger subsite-specific cohorts, and integrated translational profiling to better define which patients are most likely to benefit from induction chemoimmunotherapy.

## Conclusions

In this retrospective study of HPV-negative OPSCC, the addition of ICI to induction chemotherapy did not significantly improve PFS in the overall cohort, although numerically favorable PFS and OS trends were observed. Exploratory subgroup analyses suggested a PFS signal favoring induction chemotherapy plus ICI in patients with non-BOT primary tumors. These results support further prospective studies to determine whether primary tumor subsite may help inform patient stratification for induction chemoimmunotherapy.

## Data Availability

The raw data supporting the conclusions of this article will be made available by the authors, without undue reservation.
