# Supplementary material for: Subsite-specific benefit of induction chemoimmunotherapy in HPV-negative oropharyngeal squamous cell carcinoma
Source: Front Oncol. 2026 Jun 11;16:1861897. doi: 10.3389/fonc.2026.1861897 (PMC13293886; doi:10.3389/fonc.2026.1861897)
Supplement: Supplementary file 1 [file Table1.docx]

Supplementary Material

# Supplementary Tables

**Table S1. Antitumor activity of induction therapy in 80 patients.**

| **Response evaluation** | **Responses per RECIST by investigator assessment (n=80)** | | |
| --- | --- | --- | --- |
|  | **No. (%)** | **IC (n=57)** | **IC+ICI (n=23)** |
| Overall response rate^a)^ | 68 (85.0) | 49 (86.0) | 19 (82.6) |
| Complete response | 1 (1.2) | 0 (0.0) | 1 (4.3) |
| Partial response | 67 (83.8) | 49 (86.0) | 18 (78.3) |
| Stable disease | 6 (7.5) | 3 (5.3) | 3 (13.0) |
| Progressive disease | 6 (7.5) | 5 (8.8) | 1 (4.3) |
| Not evaluable^b)^ | 0 (0.0) | 0 (0.0) | 0 (0.0) |
| Total | 80 (100.0) | 57 (100.0) | 23 (100.0) |

Abbreviations: IC, Induction Chemotherapy; IC+ ICI, Induction Chemotherapy + ICI; RECIST, Response Evaluation Criteria in Solid Tumors, version 1.1.

a) P =0.736 by Fisher's exact test to compare response rate between IC and IC+ICI. b) Patient had no post-treatment imaging available for response assessment.

| **Table S2. Characteristics of patients (non-BOT) treated with induction therapy and PSM analysis.** | | | | | | | | | | | |
| --- | --- | --- | --- | --- | --- | --- | --- | --- | --- | --- | --- |
|  | **Before PSM** | | | |  | |  | **After PSM** | | | |
|  | **IC** | **IC+ICI** |  |  | | **IC** | | | **IC+ICI** |  |  |
| **Characteristic** | **n= 53 (%)** | **n= 17 (%)** | ***P*** | **SMD^1^** | | **n= 34 (%)** | | | **n= 17 (%)** | ***P*** | **SMD^1^** |
| **Age^2^** | 58.06 ± 9.04 | 57.65 ± 10.12 | 0.875 | 0.043 | | 57.68 ± 10.22 | | | 57.65 ± 10.12 | 0.992 | 0.003 |
| **Gender** |  |  | 1.000 | 0.076 | |  | | |  | 1.000 | 0.097 |
| Female | 5 (9.4) | 2 (11.8) |  |  | | 3(8.8) | | | 2(11.8) |  |  |
| Male | 48 (90.6) | 15 (88.2) |  |  | | 31(91.2) | | | 15(88.2) |  |  |
| **cT Stage** |  |  | 0.394 | 0.316 | |  | | |  | 1 | 0.06 |
| T1-3 | 39(73.6) | 10(58.8) |  |  | | 21(61.8) | | | 10(58.8) |  |  |
| T4 | 14(26.4) | 7(41.2) |  |  | | 13(38.2) | | | 7(41.2) |  |  |
| **cN Stage** |  |  | 0.806 | 0.163 | |  | | |  | 1 | <0.001 |
| N1-2 | 44(83.0) | 13(76.5) |  |  | | 26(76.5) | | | 13(76.5) |  |  |
| N3 | 9(17.0) | 4(23.5) |  |  | | 8(23.5) | | | 4(23.5) |  |  |
| **cTNM Stage** |  |  | 0.29 | 0.422 | |  | | |  | 0.892 | 0.167 |
| III | 15(28.3) | 2(11.8) |  |  | | 6(17.6) | | | 2(11.8) |  |  |
| IV | 38(71.7) | 15(88.2) |  |  | | 28(82.4) | | | 15(88.2) |  |  |
| Abbreviations: Non-BOT, non-base-of-tongue; IC, Induction Chemotherapy; IC+ ICI, Induction Chemotherapy + ICI; SMD, Standardized Mean Difference. | | | | | | | | | | | |
| 1. Imbalance between treatment groups was defined as a SMD ≥0.1; balance between treatment groups was defined as a SMD <0.1. 2.Variables were described by mean ± SD. | | | | | | | | | | | |

| **Table S3. Characteristics of patients (BOT) treated with induction therapy and PSM analysis.** | | | | | | | | | | | |
| --- | --- | --- | --- | --- | --- | --- | --- | --- | --- | --- | --- |
|  | **Before PSM** | | | |  | |  | **After PSM** | | | |
|  | **IC** | **IC+ICI** |  |  | | **IC** | | | **IC+ICI** |  |  |
| **Characteristic** | **n= 20 (%)** | **n= 9 (%)** | ***P*** | **SMD^1^** | | **n= 9 (%)** | | | **n= 9 (%)** | ***P*** | **SMD^1^** |
| **Age^2^** | 64.45 ± 6.26 | 63.78 ± 4.79 | 0.331 | 0.418 | | 61.56 ± 6.60 | | | 63.78 ± 4.79 | 0.426 | 0.385 |
| **Gender** |  |  | 1.000 | 0.226 | |  | | |  | 1.000 | 0.500 |
| Female | 1(5.0) | 1(11.1) |  |  | | 0(0.0) | | | 1(11.1) |  |  |
| Male | 19(95.0) | 8(88.9) |  |  | | 9(100.0) | | | 8(88.9) |  |  |
| **cT Stage** |  |  | 0.763 | 0.337 | |  | | |  | 1.000 | <0.001 |
| T1-3 | 18(90.0) | 7(77.8) |  |  | | 7(77.8) | | | 7(77.8) |  |  |
| T4 | 2(10.0) | 2(22.2) |  |  | | 2(22.2) | | | 2(22.2) |  |  |
| **cN Stage** |  |  | 0.043 | 1.067 | |  | | |  | 0.635 | 0.459 |
| N1-2 | 16(80.0) | 3(33.3) |  |  | | 5(55.6) | | | 3(33.3) |  |  |
| N3 | 4(20.0) | 6(66.7) |  |  | | 4(44.4) | | | 6(66.7) |  |  |
| **cTNM Stage** |  |  | 0.45 | 0.497 | |  | | |  | 1.000 | <0.001 |
| III | 9(45.0) | 2(22.2) |  |  | | 2(22.2) | | | 2(22.2) |  |  |
| IV | 11(55.0) | 7(77.8) |  |  | | 7(77.8) | | | 7(77.8) |  |  |
| Abbreviations: BOT, base-of-tongue; IC, Induction Chemotherapy; IC+ ICI, Induction Chemotherapy + ICI; SMD, Standardized Mean Difference. | | | | | | | | | | | |
| 1. Imbalance between treatment groups was defined as a SMD ≥0.1; balance between treatment groups was defined as a SMD <0.1. 2.Variables were described by mean ± SD. | | | | | | | | | | | |
